# A Novel Use for the Rigid Cystoscope: The Removal of Sacral Tacks after a Coloanal Anastamosis Dehiscence

**DOI:** 10.1155/2009/978038

**Published:** 2009-01-29

**Authors:** W. Mahmalji, H. Mackenzie, A. Chopada, A. Raza

**Affiliations:** ^1^Department of Urology, Ealing Hospital NHS Trust, Middlesex UB1 3HW, UK; ^2^Department of Colo-Rectal Surgery, Ealing Hospital NHS Trust, Middlesex UB1 3HW, UK

## Abstract

A 69-year-old female presented as an emergency with atrial fibrillation, which was treated with warfarin. She subsequently developed fresh rectal bleeding and after further investigations a Dukes B adenocarcinoma of the rectum was found. She subsequently underwent a low anterior resection, coloanal anastamosis and a defunctioning ileostomy. Three sterile surgical metallic tacks (pins) were inserted into the sacrum to stop brisk bleeding from the presacral venous plexus. Following discharge, she was readmitted with septic shock and a CT scan revealed a presacral fluid collection in the area surrounding the sacral tacks (pins) and an anastamotic dehiscence. The patient was not fit for further pelvic surgery to remove the tacks, so an alternative minimally invasive cystoscopic procedure was performed. The sacral tacks (pins) were removed by the urologist using a rigid cystoscope and cold cup biopsy forceps. To our knowledge, this is the first reported case in the literature.

## 1. Case Report

**Figure 1:**
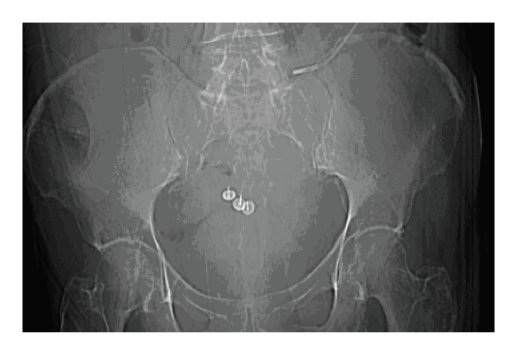
Computerised tomography (CT) scout film showing the placement of the surgical tacks in the sacrum.

A 69-year-old Caucasian female presented as an emergency in November 2006 with atrial fibrillation, which was treated with warfarin. Within one month, she developed fresh rectal bleeding. Her international normalised ratio (INR) was 2.4. Her past medical history included, type 2 diabetes, left renal calculi, and an anterior myocardial infarction in 2001 (followed by a coronary angio-bypass graft). Subsequent inpatient investigations revealed a Dukes B adenocarcinoma of the rectum 5 cm from the anal verge. She underwent a low anterior resection, coloanal anastamosis and a defunctioning ileostomy. The procedure was complicated by presacral venous plexus haemorrhage. Attempts to control the bleeding with packing and suturing were unsuccessful; three sterile surgical metallic tacks (pins) were inserted into the sacrum to compress the veins and stop the bleeding (Figure 1). This successfully controlled the haemorrhage and no transfusion was required.

After discharge she was referred back to the surgical team with hypotension and a temperature of 37.8°C. Her systolic blood pressure was consistently below 90 mmHg, and she was tachycardic with a pulse rate of 96 bpm. Examination revealed a purulent, foul smelling discharge oozing from the drain site wound. The patient was oliguric. Initial blood tests showed the following: white cell count, 22.1 × 10^9^/L (3–10 × 10^9^/L); CRP, 16 mg/L (0–5 mg/L); sodium, 131 mmol/L (135–145 mmoL/L); Potassium, 8.8 mmoL/L (3.5–5.1 mmoL/L); urea of 38.3 mmol/L (1.7–8.3 mmoL/L); creatinine of 986 umoL/L (49–92 umoL/L). Arterial blood gas sampling on room air revealed the following: pH, 7.197 (7.35–7.45); PCO_2_, 2.82 kPam; PaO2, 16.1 kPa; base excess −18.2 (−2 to 2); cHCO3 8.0.

She was admitted to the Intensive Care Unit (ICU) for haemofiltration for acute renal failure and management of her septic shock. Intravenous cefuroxime and metronidazole antibiotics were started. A CT of her abdomen and pelvis revealed a large 9.0 cm by 3.5 cm presacral collection with flecks of gas. Her abdominal drain wound swab cultured methicillin-resistant staphylococcus aureus (MRSA); linozolid was added to her antibiotic regimen.

Her discharge from ICU was followed by a transfer to the Coronary Care Unit (CCU) for palpitations and increasing shortness of breath. An echocardiogram showed moderate to severe left ventricular function and mitral regurgitation.

She continued to be treated conservatively for her pelvic collection and sepsis in CCU. Within one month she became hypotensive and was readmitted to ICU for inotropic support. Her worsening sepsis was treated with tazocin, ciprofloxacin, and metronidazole, and the presacral collection was drained under CT guidance. A rigid sigmoidoscopy showed pus around the anastamosis, and a gastrografin enema revealed an anastamotic leak. A colostomy was considered but cancelled due to her poor cardiac function and breathlessness on minimal exertion. Other contraindications to invasive surgery and a general anaesthetic included a weight loss of 3.5 stone (24.5 Kg), ongoing sepsis and recurrent urinary tract infections (UTI's). A CT Urogram (CT-IVU) was organised to investigate her renal tract and recurrent urinary tract infections, this revealed left-sided hydronephrosis and 3 cm by 1 cm presacral thick-walled collection around the region of the surgical tacks (pins) in the sacrum. Attempts were made to optimise her nutritional status and improve her sepsis prior to surgical removal of the tacks, as it was felt they may be the underlying cause of her sepsis.

**Figure 2:**
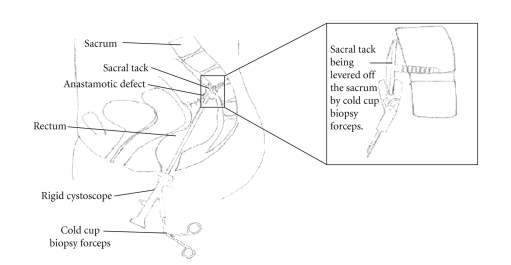
This is an illustration of the cold cut biopsy forceps passing through the anastamotic defect, and removing the tacks in the sacrum.

After a month she was still unfit for major invasive surgery or a general anaesthetic. A decision was made to remove the tacks through the rectum by passing a rigid cystoscope through the anastamotic defect and removing these from the sacrum. She underwent an examination under spinal anaesthesia. The sacral tacks (pins) were removed by the urologist using a Storz 22 French standard rigid cystoscope with a 30° lens. Normal saline was used as irrigation fluid although the view was still slightly restricted by the inflammatory tissue and some bleeding as the tacks (pins) were removed. The tacks (pins) were identified by their gold colour. The tacks (pins) were removed using a standard cold cup biopsy forceps. The edge of the circular tack (pin) was difficult to pick up with the biopsy forceps and the pin had to be levered of the sacrum by applying counter pressure on the sacrum (Figure 2). All 3 tacks were successfully removed although the patient did feel some discomfort during the procedure due to pressure on the sacrum during pin removal. There was moderate oozing from the sacrum after the tacks were removed and further haemostasis was achieved using a single strip of surgicell which was left in the cavity.

**Figure 3:**
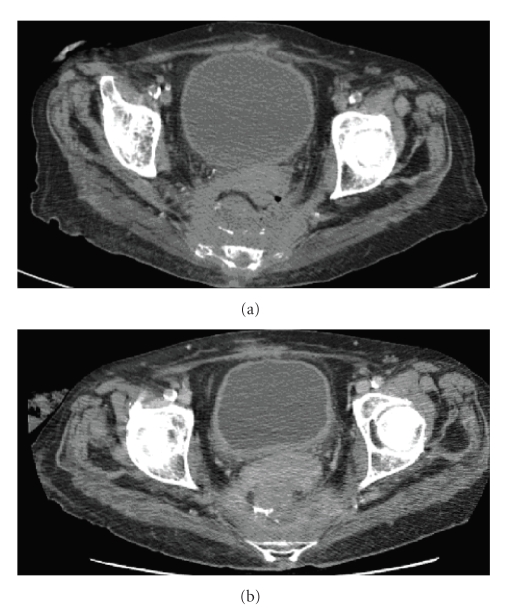
(a) CT precystoscopic tack removal. Note the thick walled presacral collection. (b) CT post cystoscopic tack removal. Although the improvement in pre-sacral soft tissue thickening and fluid collection is minimal, the patient was clinically much improved.

The patient made an excellent recovery postprocedure, with improving infective and inflammatory markers confirming resolution of the inflammatory response. The patient had a follow-up CT scan of her abdomen on 24/10/2007 and although the presacral collection improvement was minimal from a previous CT scan 4 months earlier (Figure 3), clinically the patient was much improved.

## 2. Discussion

The use of the rigid cystoscope in the anus and rectum has been described previously for closure of genitourinary fistulae [1] and the resection of rectal villous adenomas or adenocarcinomas [2, 3]. However this case is unique and is the first reported case in the literature. The use of a rigid cystoscope to remove the sacral tacks was possible using the cold cup biopsy forceps but could have been made easier in retrospect if a Collins knife or perhaps a steel wire basket had been used. This would have allowed the edge of the drawing pin (tack) to be lifted of the sacrum more readily as the tack was well embedded into the sacral bone. This case demonstrates how close cross surgical specialty referral and team work can help solve an unusual problem in a patient not fit for a general anaesthetic.
